# Glycomimetics and Glycoconjugates in Drug Discovery

**DOI:** 10.3390/ph17030323

**Published:** 2024-03-01

**Authors:** Nuno M. Xavier, Peter R. Andreana

**Affiliations:** 1 Centro de Química Estrutural, Institute of Molecular Sciences, Departamento de Química e Bioquímica, Faculdade de Ciências, Universidade de Lisboa, Ed. C8, 5° Piso, Campo Grande, 1749-016 Lisboa, Portugal; 2 Department of Chemistry and Biochemistry, School of Green Chemistry and Engineering, University of Toledo, 2801 W. Bancroft Street, Toledo, OH 43606, USA

This Special Issue of *Pharmaceuticals* presents one review and six original articles that are demonstrative of the importance of glycomimetics and glycoconjugates as privileged groups of carbohydrate-based molecules in the search for and development of bioactive substances for therapeutic/pharmaceutical purposes. Carbohydrates and glycoconjugates play important roles in biological events that are relevant for life and the progress of various diseases. The study of their structures and functions is important for developing therapeutic approaches targeting the disease-associated enzymes and events in which these molecules are implicated.

Molecular recognition processes enabling cell–cell communication or cell–pathogen recognition and adhesion are among the biological events mediated by carbohydrates through carbohydrate–carbohydrate or carbohydrate–protein interactions involving cell-surface or pathogen-surface protein/glycoproteins and glycans [1,2,3,4,5]. These events have major implications for cancer and infection, and interfering with them using glycomimetics or glycoconjugates is a promising therapeutic approach. 

In this regard, the substitution of sugar hydroxyl group(s) by a fluorine atom, a bioisoster of the latter, has been well exploited, leading to glycomimetics with the potential ability to interact and be recognized by the native carbohydrate-acting receptors or enzymes [6,7]. J. Jiménez-Barbero and co-workers report herein a new protocol for deducing the binding epitope of a ligand to a receptor and unraveling multiple binding modes through the study of the interactions between the C-type lectin DC-SIGN (Dendritic Cell-Specific ICAM-3-Grabbing Non-integrin), which is involved in viral infections, and the trifluorinated glycomimetic of the trimannoside core of mammalian glycoprotein N-glycans and its difluoro disaccharide components [Contribution 1]. The methodology used a combination of ^19^F-based STD-NMR spectroscopy methods and molecular dynamics (MD) simulations along with a created computer program that searches for the MD structures that best fit the experimental STD NMR data. 

Iminosugars are among the most studied classes of glycomimetics due to their prolific biological profile, which largely arises from their propensity for glycosidase inhibition. Their pharmaceutical significance is well demonstrated, for instance through the development of iminosugar-based molecules that became approved drugs for diabetes and for lysosomal storage diseases [8,9,10]. P. Compain and co-workers describe the synthesis and glycosidase inhibition abilities of calix [8]arene-based iminosugar clusters comprising deoxynojirimycin (DNJ) inhitopes [Contribution 2]. These clusters, designed as multivalent inhibitors, were synthesized using click-chemistry cycloaddition reaction between propargyl-DNJ derivatives and propargylated calix [8]arene and differed in the linker length between triazole and iminosugar (C6 or C9 chain), valency, and in the rigidity of the calixarene scaffold.

The best inhibitors of Jack Bean α-mannosidase (JBα-man) were found to be larger clusters, which had higher valencies and larger size and showed the best multivalent effects among the series.

In the context of developing molecules against the flavivirus tick-borne encephalitis virus (TBEV) targeting viral protein *N*-glycosylation, especially that of E protein, E. Krol and co-workers report herein the synthesis and biological evaluation of amide-, triazole-, and (triazolyl)methyl amide-linked uridine glycoconjugates, envisaged as potential inhibitors of β-1,4-galactosyltransferase [Contribution 3]. Four compounds showed potent activity against the tested TBEV strains, along with low toxicity and the ability to reduce or completely inhibit the synthesis of E protein, which mediates viral entry and induces the immune response.

The glycoconjugation of bioactive molecules or scaffolds and of bioactivity-conferring systems or moieties is a valuable strategy in drug discovery for obtaining compounds with enhanced efficacy, bioavailability, or lower toxicity as well as for tuning their selectivity and delivery. In the case of cancer cells, their low levels of oxygen (hypoxia) and nutrients as well as their higher energy demand due to inappropriate proliferation lead to an increase in glucose uptake and GLUT overexpression [11], and therefore linking glucose to a cytotoxic compound is a strategy to increase cancer-cell-selective targeting [12]. Hypoxia also plays an important role in chemotherapeutic resistance [13]. A contribution in this Special Issue by S. Agrawal and co-workers reports the synthesis and biological evaluation of a glucoconjugate derivative of the anticancer agent methotrexate [Contribution 4]. The synthesized conjugate, which contained two glucose units linked to the pyrimidine ring through an (ethyltriazolyl)methyl carbamate system, showed higher cytotoxic activity than methotrexate on cancer cells in a hypoxic microenvironment as well as higher antiproliferative and antimigration effects. 

C-Glycosyl compounds are glycoside mimetics known to exhibit diverse bioactivities which, along with their relative stability towards enzymatic hydrolysis, make them relevant glyco-scaffolds in medicinal chemistry [14,15,16]. Among them, gliflozins are anti-diabetic agents that inhibit sodium glucose cotransporter type 2 (SGLT2) [17]. Based on the general core structure of gliflozins and with the aim of identifying dual-target compounds against type 2 diabetes that inhibit SGLTs and glycogen phosphorylase (GP), the contribution of É. Bokor and co-workers reports the synthesis and biological evaluation of novel (C-β-D-glucopyranosylhetaryl)methyl arene-type compounds comprising 1,2,4- and 1,3,4-oxadiazole, pyrimidine, and imidazole motifs [Contribution 5]. Various compounds displayed low micromolar inhibition of SGLT2, aside from showing no cytotoxicity. Moreover, some previously synthesized aryl-substituted glucopyranosyl azole GP inhibitors included in this study also showed low micromolar inhibition of SGLTs, with a more potent effect on SGLT2. The best dual inhibitor was a 2-glucosyl-2-naphthylimidazole derivative with namomolar and low micromolar inhibition of GP and SGLT2, respectively.

Glycosylation is a frequently used method of post-translational modification of therapeutic proteins. The glycosylation pattern is critical for the stability, solubility, safety, immunogenicity, and pharmacokinetic properties of this category of biopharmaceuticals [18]. A contribution by I. Jongerius and co-workers reports a method for producing a recombinant glycoprotein that has a similar function in vitro and similar serum half lives in vivo to its plasma counterpart [Contribution 6]. The C1-inhibitor protein (C1-INH), which is used in the therapy of hereditary angioedema, was the model glycoprotein focused on in the work. The fully functional recombinant C1-INH with the desired glycosylation was produced in Chinese hamster ovary (CHO) cells using simple purification and screening tools. 

Peptidoglycan (PGN) is a major component of the bacterial cell wall. This complex glycoconjugate is essential for bacterial cell viability and integrity. Disturbing peptidoglycan biosynthesis is an important approach in antibiotic research [19,20,21] since it leads to a disruption of the bacterial cell wall’s integrity, leading to cell lysis. Moreover, structural elements of PGN are recognized by the human innate immune system, thus playing a key role in triggering immune responses to bacteria [22,23]. PGN comprises glycan chains of alternating N-acetylglucosamine (GlcNAc) and N-acetylmuramic acid (MurNAc) residues, cross-linked via peptide chains connected to a lactyl group at C-3 of the MurNAc moiety. The synthesis of such a complex structure and of PGN fragments is highly important for investigating their role in bacterial metabolism and in host infection/immune response and their recognition by the host, contributing to the identification of the biologically relevant fragments. In a review, M. M. Marques, S. R. Filipe, and their co-workers cover the biosynthesis of PGN and chemical/chemoenzymatic synthetic approaches for PGN fragments [Contribution 7]. The routes towards GlcNAc-MurNAc di- and oligosaccharides, di- and oligosaccharides comprising aminoacid strands linked to the MurNAc moiety, lipid derivatives, and cross-linked PGN fragments are surveyed, highlighting major synthetic challenges such as stereoselective glycosylation involving GlcNAc derivatives. 

We thank all authors as well as the reviewers for their valuable contributions to this Special Issue and hope that this interesting collection of papers inspires our readers in this exciting field of glycosciences!
